# Severe acute alcoholic hepatitis and hepatorenal syndrome: role of transjugular intrahepatic portosystemic stent shunt

**Published:** 2012-06-18

**Authors:** G Testino, S Leone, C Ferro, P Borro

**Affiliations:** * Department of Specialist Medicine, S. Martino Hospital, Genoa, Italy; ** Department of Radiology, San Martino Hospital, Genoa, Italy

**Keywords:** liver transplantation, portal hypertension, renal failure

## Abstract

Acute Alcoholic Hepatitis (AAH) is a syndrome of progressive inflammatory liver injury associated with long-term heavy intake of ethanol. Mild to moderate forms of AAH frequently respond to alcoholic abstinence, whereas severe AAH is characterized by a poor prognosis.

Up to 40% of these patients die within 6 months upon symptoms onset. This high rate of mortality is due to different factors: liver failure, severe infections, and portal hypertension with variceal bleeding and hepatorenal syndrome (HRS).

In AAH, HRS is a common complication that leads to the death of more than 90% of the patients within 3 months, unless they had been liver transplanted.

Transjugular Intrahepatic Portosystemic Stent Shunt (TIPS) has been increasingly used in the management of portal hypertension and its complications, and, it might become a valuable option in patients with HRS awaiting LT.

This study has taken into consideration 9 consecutive patients affected by severe AHH with HRS suitable for TIPS.

We have determined serum creatinine, blood urea nitrogen, serum sodium, sodium urinary excretion and urine volume in all patients, before the intervention, 7 days and 30 days after TIPS. Seven patients were transplanted within 6 months.

After TIPS, the renal function improved with significant reduction in serum creatinine and increase in urine volume.

We can conclude that TIPS is a valuable option in patients with severe AAH complicated by HRS and are waiting for liver transplantation.

## Introduction

Acute Alcoholic Hepatitis (AAH) is a syndrome of progressive inflammatory liver injury associated with long-term heavy intake of ethanol. It is characterized by variable laboratory and clinical findings: jaundice, prolonged prothrombin time, liver failure, ascites, hepatic encephalopathy [**1,2**].

In AAH, hepatorenal syndrome (HRS) is a common complication that leads to the death of more than 90% of the patients within 3 months, unless they had been liver transplanted (LT) [**3**]. 

Liver transplantation (LT) is the cornerstone of treatment, since renal dysfunction is usually reversible with the correction of the underlying cause (portal hypertension). 

However, because of the limited survival of the patients with SAH and HRS and the limited availability of organs, only a small percentage of patients can actually reach LT.

Type-2 HRS is a moderate, steady renal impairment. It arises spontaneously and it is the main underlying mechanism of refractory ascites.

Type-1 HRS is a rapidly progressive renal failure that is defined as the doubling of serum creatinine reaching a level >2.5 mg/dl in less than two weeks. A precipitating factor is frequently identified in type 1 HRS.

Transjugular Intrahepatic Portosystemic Stent Shunt (TIPS) has been increasingly used in the management of portal hypertension and its complications, and, it might become a valuable option in patients with HRS awaiting LT [**4**].

## Patients and methods

This study has considered 9 consecutive patients (6 males, 3 females, average age of 49 years old) affected by severe AAH with HRS (HCV and HBV negative) [**5**] (Child-Pugh score between 10 and 12, MELD – Model for End-Stage Liver Disease – score > 21) suitable for TIPS. 

The diagnosis of severe AAH was histologically ascertained by transjugular liver biopsy. 

A diagnosis of HRS was made after excluding the other possible causes of renal function deterioration, on the basis of the International Ascites Club, criteria such as low glomerular filtration rate (as indicated by serum creatinine of >1.5 mg/dl or 24- hour creatinine clearance <40 ml/min), absence of shock, ongoing bacterial infections and current or recent treatment with nephrotoxic drugs, absence of fluid losses, no sustained improvement in renal function after plasma volume expansion, proteinuria lower than 500 mg/day, no ultrasonographic evidence of obstructive uropathy or parenchymal renal disease. Moreover, all the patients had low urine volume, very low urine sodium (< 10 mEq/l) and urine osmolality greater than the plasmatic one [**4,6**].

All the patients had type-1 HRS, characterized by a rapidly progressive renal failure that is defined by the doubling of the initial serum creatinine to a level > 2.5 mg/dl or by 50% reduction in creatinine clearance to a level < 20 ml/min in less than 2 weeks.

2 out of the 9 patients included in our study had an episode of Spontaneous Bacterial Peritonitis (SBP) before the development of HRS, which has been treated and solved at least 1 week before the inclusion. Renal function deterioration in these two patients was rapidly progressive despite the resolution of the infection. This type of renal impairment after a SBP is not spontaneously reversible and it leads to a patients’ rate of death of 100%. No precipitating factors were found in the other seven patients. Four patients had refractory ascites before the development of type-1 HRS. 

Written informed consent was obtained from all the patients before TIPS intervention.

Exclusion criteria included ongoing infections, hepatocellular carcinoma and other malignancies, symptomatic cardiac or respiratory diseases or recent gastrointestinal bleeding (in the last week).

A therapeutic paracentesis followed by intravenous administration of albumin (8 gr/l), was performed in all patients before TIPS insertion. 

After mild intravenous sedation and analgesia (meperidine and midazolam), a puncture needle was advanced through the transjugular, in a catheter, through the inferior cava into one of the three hepatic veins. Subsequently an intrahepatic branch of the portal vein was punctured and the shunt was established by the implantation of Wallstent (Boston Scientific, Natick, USA).

Serum creatinine, blood urea nitrogen (BUN), serum sodium, sodium excretion and urine volume were determined in all patients before TIPS, and 7 and 30 days after TIPS.

The statistical analysis of the results was made by using paired Student’s test and ANOVA with Dunn’s test and Bonferroni comparison. Results are presented as means ± SEM. P < 0.05 was considered statistically significant.

## Results

All stents were successfully established. Complications occurred in 4 patients: 3 of them had a temperature above 38°C and 1 of them experienced vomiting after the procedure.

Causes of death were septicaemia in 1 patient and liver failure in another one. Seven patients were transplanted within 6 months. 

No patients developed hepatic encephalopathy resistant to medical treatment.

The results concerning the renal parameters are listed in Table 1; they indicate an improvement of renal function.

30 days after TIPS insertion, an ultrasonographic intermediate examination was performed in all patients evidencing detectable ascites without the need for paracentesis.

**Table 1:** Function renal parameters before and after TIPS

	Baseline	7 days	30 days	p
Serum Creatinine (mg/dL)	5.2+/-0.9	3.6+/-1.1	1.6+/-0.6	.04
BUN (mg/dL)	110+/-8	109+/-7	51+/- 11	.007
Serum sodium (mEq/L)	125+/-4	123+/3	135+/- 4	n.s.
Urine sodium (mEq/d)	2.5+/- 0.5	7+/- 3	10+/-3	n.s
Urine volume (mL/d)	250+/-40	840+/-170	1100+/-210	.003

## Conclusions

It is well known that TIPS insertion improves renal dysfunction in chronic liver disease. Patients with poor renal function are those who benefit the most from TIPS implantation.

There are no studies in literature, which evaluate the clinical effects of TIPS in severe AAH patients with HRS.

A reasonable approach is to treat the initial event underlying the syndrome: the circulatory dysfunction that leads to portal hypertension. So, it is important to obtain normal portal pressure values [**4,7,8**].

The development of TIPS has introduced the idea of treating HRS by reducing portal pressure [**5**].

Our beneficial results could be due to the obtainment of an improved filling of the central venous system. This fact causes a decrease in the production of neurohumoral vasoconstrictor mediators and, consequently, a reduction of sinusoidal pressure. Decreased values of sinusoidal pressure relieve sodium retention, down regulating the direct sympathetic nervous system effects on the proximal tubule.

In the present era of organs shortage, the pre-transplant management of HRS will assume an increasingly important role. We have observed that the setting of TIPS permits an improvement in the sodium levels and in the renal function parameters. It is, in fact, well known that a reduction of serum sodium levels and an increase of serum creatinine values influence the pre and post-LT course and can modify the mortality rate of the patients’ list [**9,10**].

Because of the almost universally fatal outcome of severe AAH associated with type-1 HRS, TIPS may become a valuable option for patients awaiting liver transplantation, after at least 3/6 months of alcohol abstinence.

## References

[1] Stickel  F, Seitz  HK (2010). Alcoholic steatohepatitis. Best Practice Res Clin Gastroenterol.

[2] Negreanu  L, Tribus  LC, Purcarea  M (2011). Spontaneous esophageal mucosal dissection in a patient with upper digestive bleeding and esophageal varices. . J Med Life.

[3] Testino  G (2008). Alcoholic diseases in hepato-gastroenterology: a point of view. Hepato-Gastroenterology.

[4] Testino  G, Ferro  C, Sumberaz  A (2003). Type-2 hepatorenal syndrome and refractory ascites: role of transjugular portosystemic stent shunt in eighteen patients with advanced cirrosi awaiting orthotopic liver transplantation. Hepato-Gastroenterology.

[5] Ansaldi  F, Bruzzone  B, Testino  G (2006). Combination hepatitis C virus antigen and antibody immunoassay as a new tool for early diagnosis of infection. J Viral Hepat.

[6] Arroyo  V, Gines  P, Gerbes  AL (1996). Definition and diagnostic criteria of refractory ascites and hepatorenal syndrome in cirrhosis. Hepatology.

[7] Salerno  F, Gerbes  A, Gines P (2007). Diagnosis, prevention and treatment of hepatorenal syndrome in cirrhosis. Gut.

[8] Testino  G, Ferro  C (2010). Hepatorenal syndrome: a review. Gastroenterology.

[9] Anderson  CL, Saad  WEA, Kalagher  SD (2010). Effect of transjugular intrahepatic portosystemic shunt placement on renal function: a 7-year, single-center experienc. J Vasc Intervent Radiol.

[10] Sumberaz  A, Centenaro  M, Ansaldi  F (2007). Relationship between laboratory parameters and intensive care unit stay post-liver transplantation: proposal of a model. Transplant Proc.

